# A Cautionary Tale: Persistent Trigeminal Artery and Wada Test

**DOI:** 10.1177/08830738251357080

**Published:** 2025-07-21

**Authors:** Ranjith Kumar Manokaran, Samyami Sangeeta Chowdhury, Prakash Muthusami, Suvasini Sharma, Elizabeth Kerr, Shelly Weiss, Puneet Jain

**Affiliations:** 1 Division of Neurology, Department of Paediatrics, 7979 The Hospital for Sick Children, University of Toronto, Toronto, Canada; 2 Department of Neurology, Sri Ramachandra Institute of Higher Education and Research, Chennai, India; 3 Department of Diagnostic Imaging & Image-Guided Therapy, 7979 The Hospital for Sick Children, University of Toronto, Toronto, Canada; 4 Department of Psychology, 7979 Hospital for Sick Children, University of Toronto, Toronto, Canada

**Keywords:** epilepsy surgery, etomidate, etomidate speech test, stroke

**Figure 1.**
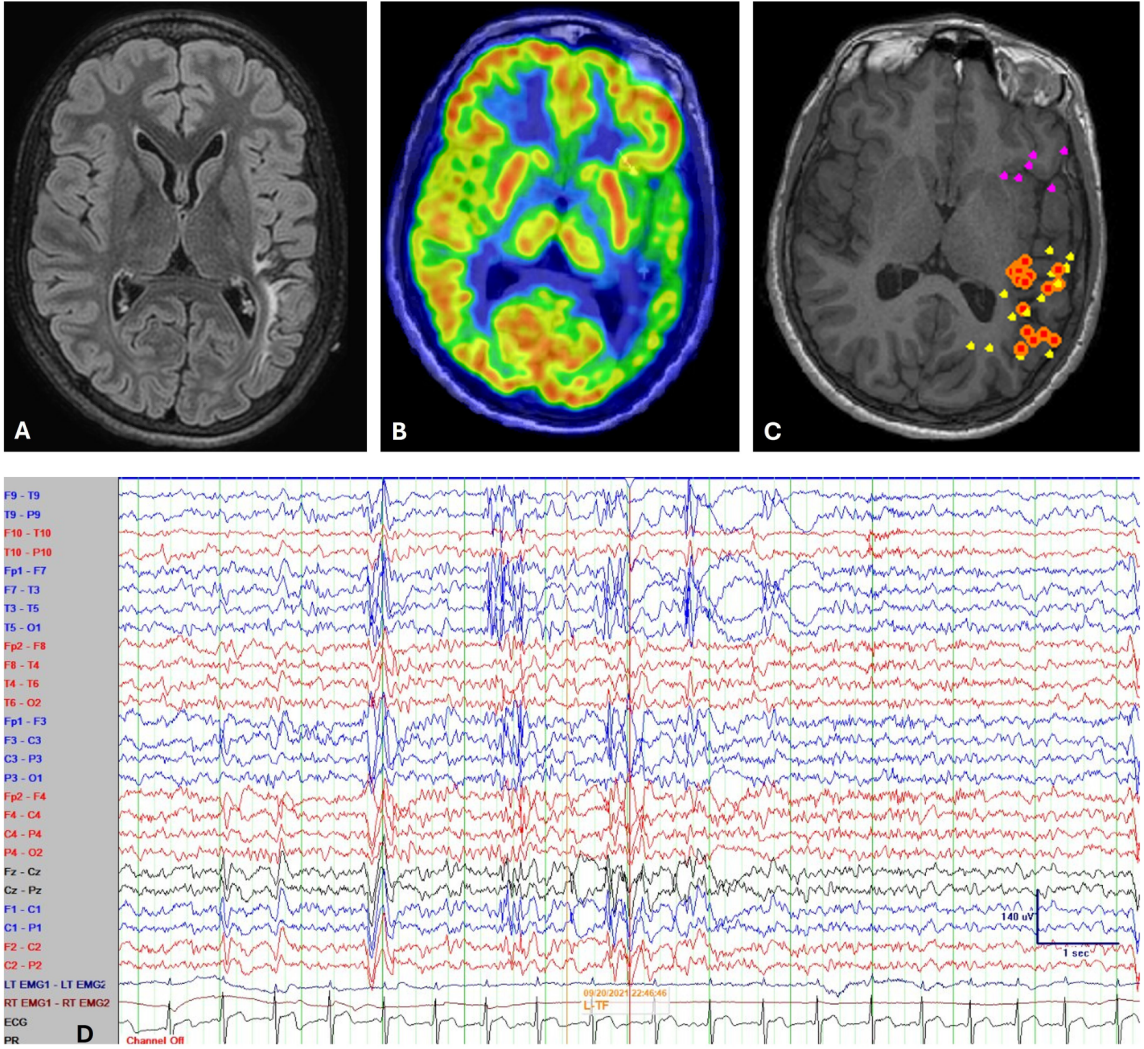
Radiology and Neurophysiology of the Reported Patient. (A) Axial FLAIR (Fluid-Attenuated Inversion Recovery) MRI Sequence Showed Encephalomalacia in the Left Temporoparietal Region. (B) Fluorodeoxyglucose Positron Emission Tomography (PET) Coregistered to MRI Showed Hypometabolism in the Concordant Region. (C) MRI Showed Spike Dipole Cluster Over Left Temporo-Occipital Junction (Online image - Orange Dots), and Scattered Dipoles Over Left Parietal (Online image - Yellow) and Left Inferior Frontal Region (Online image - Pink Dots). (Note That Since All Dipoles Have Been Projected on to the 2-Dimensional Figure, Some of Them May not Represent Their True Locations in a 3-Dimensional Space.) (D) Interictal EEG Showed Polyspike and Waves Mixed with Polymorphic Delta/Theta Activity Over the Left Hemisphere, More Prominent in the Temporal Regions. Bipolar Longitudinal EEG Montage, High-Frequency Filter 70 Hz, Low-Frequency Filter 1 Hz, Sensitivity 7 μV/mm, Time Base 30 mm/s, and Sampling Rate 1 kHz. MRI, Magnetic Resonance Imaging.

A 14-year-old right-handed boy presented at 1.5 years of age with vigabatrin-responsive infantile epileptic-spasm syndrome. He later developed drug-resistant seizures (weekly focal impaired awareness seizures at 6 years of age and daily myoclonic seizures at 10 years). He also had mild intellectual disability and attention-deficit hyperactivity disorder (ADHD). His MRI showed left temporoparietal encephalomalacia due to presumed perinatal ischemic stroke. Positron emission tomography and magnetic resonance imaging (MRI) were concordant (Figure 1, A-C). Ictal and interictal electroencephalography (EEG) were predominantly generalized, with left-temporal localization (Figure 1D). Functional MRI for language showed bilateral language representation. Chromosomal microarray analysis and a next-generation sequencing epilepsy gene panel test was nondiagnostic.

**Figure 2.**
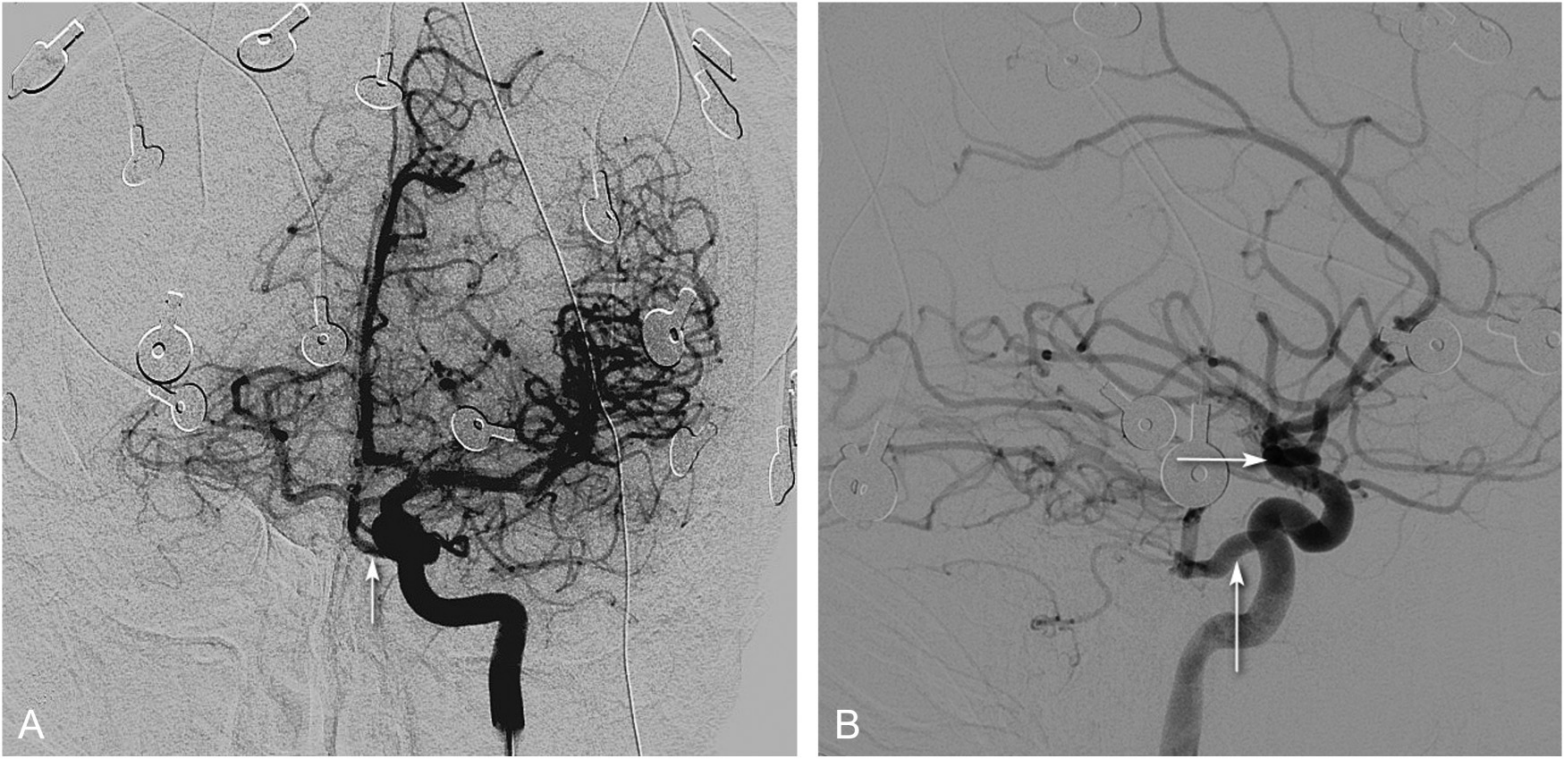
Catheter-Directed Left Internal-Carotid Angiography Pre–Wada Test. (A) Frontal Projection: Persistent Trigeminal Artery (White Arrow) Communicating With The Midbasilar Artery and Filling The Posterior-Cerebral Arteries. (B) Lateral Projection: Large Persistent Trigeminal Artery (Vertical Arrow) Arising From The Cavernous Segment of Internal Carotid Artery. Note The Absence of Posterior Communicating Artery (Horizontal Arrow).

A Wada test (etomidate speech test [EST]) was planned. However, pre–etomidate speech test the left internal carotid injection on catheter angiography demonstrated primitive persistent trigeminal artery anastomosing with the midbasilar artery (Figure 2). Thus, the procedure was aborted. He is being planned for stereo-EEG evaluation for delineating epileptogenic zone and language mapping.

**Figure 3.**
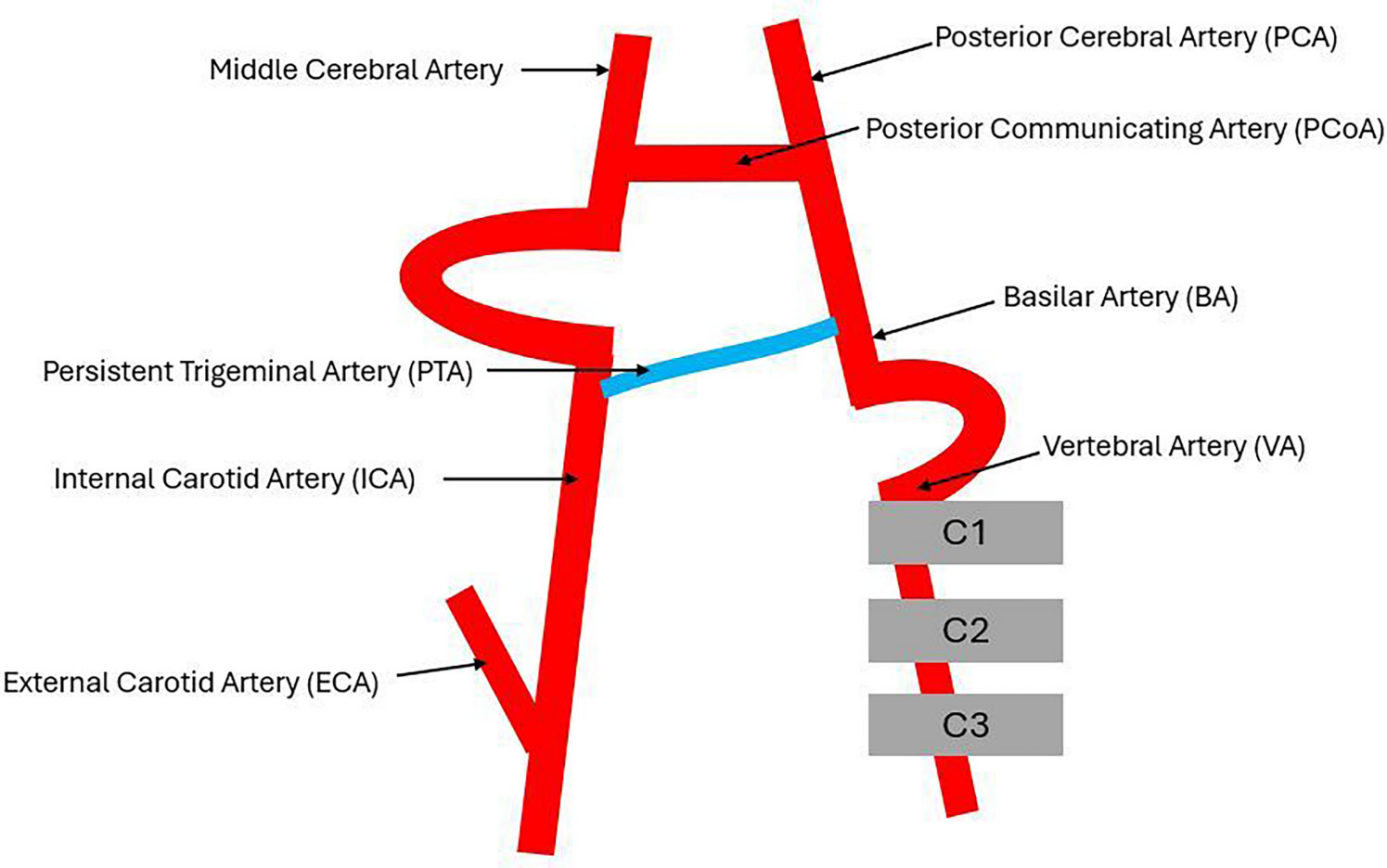
Schematic Diagram to Show The Relationship of The Persistent Trigeminal Artery (PTA) to The Internal Carotid Artery (ICA) and Basilar Artery (BA).

Persistence of primitive intracranial embryonic anastomoses occurs in 0.1% to 1.25% of the population, persistent trigeminal artery being the most common.^1^ The persistent trigeminal artery is an embryonic remnant between the proximal intracavernous segment of the internal carotid artery and the middle to distal portion of the basilar artery (Figure 3).^2^ The role of the persistent trigeminal artery in utero is to supply the basilar artery before the posterior-communicating and vertebral arteries have developed. The origin may be more proximal in some cases and therefore misinterpreted as the otic artery, which is another rare embryonic arterial remnant. The Saltzman classification system describes 3 types of persistent trigeminal artery^3^:

Type I: the persistent trigeminal artery supplies the distal vertebrobasilar arteries, the posterior communicating artery is absent, and the caudal basilar artery may be either absent or hypoplastic with the presence of hypoplastic distal vertebral arteries.

Type II: the persistent trigeminal artery supplies the superior cerebellar arteries with the posterior cerebral arteries being supplied by the posterior communicating artery.

Type III: this type describes how the persistent trigeminal artery terminates. It does not join the basilar arteries but terminates in the superior cerebellar artery (type IIIa), anterior inferior cerebellar artery (type IIIb) or posterior inferior cerebellar artery (type IIIc).

The newer classification systems by Weon et al propose a more comprehensive classification with 5 subtypes, where type IV describes the persistent trigeminal artery supplying the ipsilateral posterior communicating artery and type V is analogous to the 3 Saltzman variants (IIIa-IIIc).

The persistent trigeminal artery may be prone to aneurysms because of its bifurcation.^1^ There is an association with ischemic stroke, albeit less evidence for vertebrobasilar insufficiency. Adult case reports have described a variety of ischemic events with persistent trigeminal artery and posterior circulation infarcts.^4^ Persistent trigeminal artery is seen in vasculopathy syndromes such as PHACE syndrome (Posterior fossa brain malformations, Hemangiomas [typically large facial hemangiomas], Arterial anomalies, Cardiovascular anomalies, Eye abnormalities, and Sternal defects). There is a lack of evidence to demonstrate an association of persistent trigeminal artery, with ischemic infarct in the pediatric PHACE syndrome population. However, the risk of embolic occurrences is seen in context of anomalies in addition to persistent trigeminal artery.^1^

Thrombosis of the persistent trigeminal artery is a rare occurrence, although it may occur in the context of an internal carotid artery dissection.^1^ In patients with persistent trigeminal artery and vertebrobasilar hypoplasia, this causes reduced vascular supply to the posterior fossa; therefore, propensity to ischemic events and occurrence of steal phenomenon. Poor antegrade flow from atretic vertebral arteries may contribute to posterior fossa ischemia. Given the serious risk of anesthetizing the brainstem, it is pertinent to identify a persistent trigeminal artery prior to Wada test.^4^
